# Assessment of microbiome changes after rumen transfaunation: implications on improving feed efficiency in beef cattle

**DOI:** 10.1186/s40168-018-0447-y

**Published:** 2018-03-27

**Authors:** Mi Zhou, Yong-Jia Peng, Yanhong Chen, Christen M. Klinger, Masahito Oba, Jian-Xin Liu, Le Luo Guan

**Affiliations:** 1 Department of Agricultural, Food and Nutritional Science, University of Alberta, 416F Agr/For Centre, Edmonton, Alberta T6G 2P5 Canada; 2 College of Animal Sciences, Zhejiang University, Hangzhou, 310058 Zhejiang China

**Keywords:** Adaptation, Beef cattle, Transfaunation, Rumen microbiota

## Abstract

**Background:**

Understanding the host impact on its symbiotic microbiota is important in redirecting the rumen microbiota and thus improving animal performance. The current study aimed to understand how rumen microbiota were altered and re-established after being emptied and receiving content from donor, thus to understand the impact of such process on rumen microbial fermentation and to explore the microbial phylotypes with higher manipulation potentials.

**Results:**

Individual animal had strong effect on the re-establishment of the bacterial community according to the observed profiles detected by both fingerprinting and pyrosequencing. Most of the bacterial profile recovery patterns and extents at genus level varied among steers; and each identified bacterial genus responded to transfaunation differently within each host. *Coriobacteriaceae*, *Coprococcus*, and *Lactobacillus* were found to be the most responsive and tunable genera by exchanging rumen content. Besides, the association of 18 bacterial phylotypes with host fermentation parameters suggest that these phylotypes should also be considered as the regulating targets in improving host feed efficiency. In addition, the archaeal community had different re-establishment patterns for each host as determined by fingerprint profiling: it was altered after receiving non-native microbiome in some animals, while it resumed its original status after the adaptation period in the other ones.

**Conclusions:**

The highly individualized microbial re-establishment process suggested the importance of considering host genetics, microbial functional genomics, and host fermentation/performance assessment when developing effective and selective microbial manipulation methods for improving animal feed efficiency.

**Electronic supplementary material:**

The online version of this article (10.1186/s40168-018-0447-y) contains supplementary material, which is available to authorized users.

## Background

The symbiotic microbiota in the rumen facilitates the digestion by decomposing the ingesta and degrading the plant materials into different volatile fatty acids (VFAs), ammonia, etc., to supply the host with nutrient and energy. Therefore, the improvement of rumen microbial digestion capability can possibly lead to enhanced beef/dairy feed efficiency and thus productivity. Various factors have been incorporated into previous practices to redirect rumen microbiota. For example, changing animal diets [1], reducing rumen pH [2], depleting the rumen protozoa [3], etc. have been tested in different studies. However, none of the above methods have shown consistent positive effects, indicating that the long-term effective methods to improve animal performance by manipulating rumen microbiota have not been achieved yet.

Microbial transplantation has been proposed as one of the promising methods reshaping the symbiotic microbiota and changing host performance in small animals [4–7] and human [8]. To date, only one study has been explored in dairy cows, where whole rumen content of the two donor cows was introduced to other two recipient animals [9]. However, the fermentation parameters of the recipient cows resumed to their original status soon after transplantation [9] and their bacterial profiles returned to the original status, indicating host may strongly impact on the rumen microbiota re-establishment.

Residual feed intake (RFI) is one of the measures for feed efficiency [10]. Animals with low RFI (L-RFI) value (negative) consume less feed than expected and are considered efficient, while animals with high RFI (H-RFI) value (positive) consume more feed and are designated inefficient [11]. Compositional variations of the rumen microbiota including bacteria [12–15] and methanogens [16, 17] have been observed between efficient and inefficient cattle, and this trend was also identified in cattle with different breeds and offspring from different sire breeds [12, 18]. We hypothesized that introducing rumen microbiome of efficient animals to inefficient animals may transfer the favorable traits from L-RFI animals to H-RFI animals, thus to improve the host performance. Therefore, the current study aimed to examine the extent and speed of bacterial and archaeal community establishment after exchanging rumen content between animals with different feed efficiency.

## Methods

### Animal experiment and sampling

All of the animals were cared for following the guidelines of the Canadian Council of Animal Care (Ottawa, ON, Canada), and the animal study was proved by Animal Care Use Committee Livestock, University of Alberta (Protocol No. AUP00000116). One hundred crossbred steers were fed with a feedlot diet at the Roy Berg Kinsella Research Ranch, University of Alberta, for a 3-month period, during which feed intake of individual animals was measured using GrowSafe system for RFI evaluation following Nkrumah et al. [19]. The animals with the highest RFI (*n* = 10) and lowest RFI (*n* = 10) (8 months of age) were selected and transported to the Metabolic Unit at the University of Alberta for the further rumen transplanting experiment. The cattle were fed with a diet including 56.7% of dry-rolled barley grain, 28.3% of dry-rolled oats grain, 10% of alfalfa pellet, and 5% of Premix of mineral, vitamin, and monensin. Rumen cannulation was performed after the animals adapted to the environment in the Metabolic Unit at 9–10 months of age. After recovered from the surgery, the animals were trained to get used to interacting with humans and undergoing the sampling processes. Two steers with L-RFI were removed from the study due to their discomfort with human handling. The remaining 18 steers were used for the following trials.

Eight weeks after the cannulation, the animals were separated into two groups and the experiment was done for each group a week apart. Each group contained nine animals (4 L-RFI and 5 H-RFI), among which one H-RFI steer was set as control without exchange, and the rest of the steers were paired based on comparable body weight and allocated into four transfaunation types (Table 1). Specifically, LL referred to L-RFI animals receiving content from L-RFI donors; LH referred to L-RFI animals receiving content from H-RFI donors; HL referred to H-RFI animals receiving content from L-RFI donors; HH referred to H-RFI animals receiving content from H-RFI donors. The contents of the steers of each pair were exchanged 2–3 h prior to feeding. On the day of exchange, the rumen contents were firstly completely removed from all animals except the control, then the rumen was rinsed with 30 L of sterile pre-warmed phosphate-buffered saline (PBS, pH 6.8) for at least three times until the solution was colorless. Lastly, the rumen contents were transferred to between animals within the assigned pair. To avoid the adverse effects of oxygen exposure to the rumen microbiome, four people worked together for the rinse and exchange procedure, ensuring the whole process was processed within 15 min for each animal. Detailed steps were explained in Additional file 1. Rumen digesta including liquid and solid was collected from four rumen locations (cranial, caudal, top, and bottom) consistently 1–1.5 h before feeding for three consecutive days (D-2 to D0 and D26–D28) for rumen fermentation parameter measurements as described by Schlau et al. [20]. Whole rumen digesta mixed from the samples collected from the four rumen locations as stated above were also collected from the rumen cannula on day 0, just before exchange, and after the exchange on days 1, 3, 7, 14, and 28, for microbial and VFA profiling assessment. The samples were placed on dry ice immediately and transferred to − 80 °C for storage until being processed.

**Table 1:** Animal information and transplantation pair design

Group	Exchange pair	Animal ID	Exchange type (recipient RFI-donor RFI)	RFI (measured before selection)	Body weight before transfaunation (kg)
1	9/231	9	LL	− 0.95 (L)	488
		231	LL	− 1.11 (L)	459
1	31/107	31	LH	− 0.91 (L)	480
		107	HL	1.99 (H)	468
1	485/463	485	HL	0.78 (H)	405
		463	LH	− 1.08 (L)	459
1	223/135	223	HH	1.03 (H)	447
		135	HH	0.86 (H)	458
1	Control	73	–	1.14 (H)	469
2	201/247	201	LL	− 1.75 (L)	356
		247	LL	− 1.18 (L)	361
2	483/481	483	LH	− 0.73 (L)	398
		481	HL	0.99 (H)	417
2	67/89	67	HL	1.68 (H)	495
		89	LH	− 1.25 (L)	446
2	59/35	59	HH	1.68 (H)	496
		35	HH	1.55 (H)	472
2	Control	169	–	0.81 (H)	412

### Ruminal fermentation measurements

Rumen pH was measured for three consecutive days before each exchange following Penner et al. [21]. Rumen fluid samples (obtained after filtration by cheese cloth) were subjected to VFA profiling using gas chromatography: Briefly, samples were injected by an auto sampler (Model 8200, Varian Incorporated; Walnut Creek, CA) into and run on Stabilwax-DA column (30 m × 0.53 mm i.d. × 0.5 μm film, Restek Corporation; Bellefonte, PA) on a Varian Gas Chromatographer (Model 3400) for measurements following the identical settings described by Schlau et al. [20]. Ammonia-nitrogen was determined using colorimetric procedure by measuring the absorbance at 600 nm following Schlau et al. [22]. Dry matter intake (DMI) of each animal was recorded before experiment and during the period of transfaunation with identical steps in Schlau et al. [20].

### Bacteria and archaea quantification

Quantitative real-time PCR was applied to estimate the bacterial and archaeal populations by measuring the copy numbers of bacterial and archaeal 16S rRNA genes with the universal primer pair U2 [23] and archaea universal primer pair uniMet1-F/R [16], respectively. The standard curves for quantification were generated according to Li et al. [24] for total bacteria quantification and Zhou et al. [16] for total methanogen quantification respectively. The quantity of the copy numbers was obtained based on the plotted standard curves generated by StepOne™ software (V2.1, Applied Biosystems, Foster City, CA). The final copy number of total bacteria and archaea 16S rRNA gene per gram of rumen content was calculated following previous studies [13, 16].

### DNA extraction and PCR-DGGE profiling of bacteria and archaea

Total DNA was extracted from each rumen content sample using beads beating and phenol-chloroform extraction as described previously [12]. PCR amplification was performed on each sample respectively, and the products for the four locations of each animal were combined. The V2-V3 region of the bacterial 16S rRNA gene was amplified with bacterial universal primers (HDA1-GC and HDA2), and the V2-V3 region of the archaeal 16S rRNA gene was amplified with archaeal universal primers (GC-ARC344f/519r). Bacterial and archaeal 16s rRNA gene PCR amplicons were subjected to denaturing gradient gel electrophoresis (DGGE) analysis following Hernandez-Sanabria et al. [13] and Zhou et al. [17], respectively. The PCR-DGGE profiles of day 0, 1, 3, 7, and 28 rumen samples were selected to assess the short-term microbial adaptation patterns (D0–D7) and to evaluate the final recovery status (D0 vs. D28). All of the PCR-DGGE profiles were analyzed with the BioNumerics software package (V6.0, Applied Maths, Austin, TX). Similarity of bacterial profiles were determined using the Dice similarity coefficient (*D*_sc_), with cluster dendrograms generated using the unweighted pairwise grouping method with mathematical averages (UPGMA) clustering algorithm with 1% position tolerance.

### Pyrosequencing analysis of bacterial recovery patterns

To compare the bacterial recovery patterns thoroughly, DNA samples of D0, D1, D7, and D28 were selected based on PCR-DGGE analyses, to reflect both short-term (D0–D7) and long-term changes (D0 vs. D28) but also to reflect the progress of the changes. The samples of these four days were subjected to pyrosequencing analysis. Each DNA sample was diluted to 50 ng/μl template to amplify partial bacterial 16S rRNA gene fragments (V1-V3 region) with primer A-338 (5′-TGCTGCCTCCCGTAGGAGT-3′)/primer B (5′-AGAGTTTGATCCTGGCTCAG-3′) [25]. The reaction system (50 μl) included 1 μl of template, 1 μl of 10 mM deoxynucleoside triphosphate, 2.5 U of Taq polymerase (Invitrogen, Carlsbad, CA), 1× PCR buffer, 1 μl of 50 mM MgCl_2_, 1 μl of 20 pmol of each primer, and nuclease-free water. The reaction program was an initial denaturation for 5 min at 95 °C; 30 cycles at 95 °C for 30 s, 53 °C for 30 s, and 72 °C for 1 min; and a final elongation for 7 min at 72 °C. The amplicons were run on a 1.2% agarose gel, the bands of proper size (~ 400 bp) were excised, and the DNA were extracted from the bands using QIAEX II gel extraction kit (Qiagen Sciences, MD). The concentration and quality of the eluted amplicons were measured using ND-1000 spectrophotometer (NanoDrop Technologies, Wilmington, DE). Pooled sample containing 25 ng of each purified amplicon was sent to GenomeQuebec (Montreal, QC) for pyrosequencing analysis using 454 Titanium FLX (Roche). The reads were processed using Quantitative Insights into Microbial Ecology (QIIME) program (1.9.0) [26] to evaluate the changes in the microbial community. Taxonomic analyses assigned the reads to different OTUs at phylum, class, order, family, and genus level based on the SILVA database (SILVA128) [27] with Uclust method, with chimera check and singleton removed. The species richness of each sample was estimated with Chao1 index with 97% sequence similarity, and the OTU numbers were assigned based on unique OTU reads. Shannon index and Simpson index were calculated to indicate community diversity through QIIME. To avoid missing the phylotypes identified from the samples [28], OTUs from each sample were only normalized with the lowest number identified from the entire sample set prior to analyzing the beta diversity. Common and unique OTUs were analyzed with Venny’s online tool [29]. Detailed scripts and settings were listed in Additional file 1. Both the data from control group and the transfaunation group were performed following the same procedures. Changes of microbial phylotypes were analyzed for each transfaunation type separately at different phylogenic levels.

### Co-variation between microbiome and feed efficiency for individual animals

The differences of microbial profiles were defined by profile distances, where 0 < d ≤ 0.25 indicated highly similar, 0.25 < d ≤ 0.50 indicated similar, 0.50 < d ≤ 0.75 indicated dissimilar, and d > 0.75 indicated highly dissimilar. The difference of feed efficiency was defined by |ΔFCR|, where |ΔFCR| < 3 was considered stable and |ΔFCR| > 10 was considered changed significantly. The microbial profile distance and ΔFCR were compared before and after transfaunation procedure for each individual animal.

### Statistical analysis

Similarity of the obtained profiles was analyzed with Analysis of Similarity (ANOSIM) program run within R statistics (http://www.R-project.org) and plotted with UPGMA and PCoA methods. Correlation between bacteria population and fermentation parameters was evaluated using PROC CORR within SAS (V9.2, SAS Institute Inc., Cary, NC), with bacteria numbers as independent variable and all fermentation measures as dependent variables. To avoid type 1 error, only the microbial phylotypes occurred in at least 12 animals (75% of samples) were subjected to the correlation analyses. Effects of exchange type, adaptation patterns, and animal pair on dry matter intake (DMI), average daily gain (ADG), feed conversion ratio (FCR), VFAs, ammonia nitrogen, pH, and microbial abundance were evaluated with analysis of variance (ANOVA) within SAS (version 9.2). The statistical model included fixed effects of exchange type/adaptation patterns/animal pair and random effects of animal group and animal nested within group. Significance was defined with *P* < 0.05, and trend was defined with 0.05 ≤ *P* < 0.1. Analyses about the transfaunation effects on each identified microbial phylotype were only performed for those observed in more than 50% of the group of animals. Significance was defined with FDR value < 0.1, and trend was defined with 0.1 ≤ FDR value < 0.2.

## Results

### Fermentation parameters, feed efficiency parameters, and microbial population in steers after microbial transplantation

To evaluate how the microbial composition change impacted the microbial fermentation, and thus host performance (DMI, ADG, FCR), the rumen fermentation parameters including VFA profiles, ammonia, and mean pH of the animals before (D-2 to D0) and after (D26 to D28) the transfaunation were compared among the four exchange types. As shown in Table 2, most of the measurements did not change after the animals receiving non-native rumen contents, regardless of RFI classification of the donor and recipient. The only noticeable change was observed for the archaea abundance, where the population increased after the transfaunation in three out of four exchange types (HL/LH/LL). Among all of the measurements, DMI (*R* = 0.500, *P* = 0.048) and total VFA (*R* = 0.604, *P* = 0.013) were correlated before and after the transfaunation, while others did not show correlation before and after the transfaunation process (Additional file 1: Table S1).

**Table 2:** Comparison of rumen parameters and microbial population among transfaunation types

		Period	HH (*N* = 4)	HL (*N* = 4)	LH (*N* = 4)	LL (*N* = 4)
VFAs	Total VFA, mM	Before	124.1 ± 7.2	156.2 ± 9.5	127.6 ± 7.2	123.2 ± 12.5
		After	147.7 ± 10.6	165.5 ± 10.3	144.1 ± 5.4	146.6 ± 14.4
		*P*	*	NS	NS	NS
	Acetate, mol/100 mol VFA	Before	53.1 ± 2.3	50.3 ± 2.7	50.0 ± 3.4	51.2 ± 2.7
		After	47.0 ± 2.6	44.1 ± 3.4	48.5 ± 3.1	49.3 ± 0.5
		*P*	†	†	NS	NS
	Propionate, mol/100 mol VFA	Before	29.7 ± 5.0	37.1 ± 5.3	33.3 ± 5.3	35.0 ± 6.2
		After	35.3 ± 3.9	40.9 ± 4.8	33.9 ± 4.1	37.6 ± 1.4
		*P*	NS	NS	NS	NS
	Butyrate, mol/100 mol VFA	Before	11.8 ± 3.1	7.4 ± 1.6	11.9 ± 2.4	9.7 ± 3.4
		After	12.2 ± 2.6	10.0 ± 1.4	12.9 ± 1.8	8.9 ± 1.1
		*P*	NS	NS	NS	NS
N-NH_3_	Ammonia, μg/ml	Before	10.5 ± 2.5	10.2 ± 1.5	13.9 ± 2.0	10.8 ± 3.6
		After	8.3 ± 1.2	7.0 ± 1.5	12.3 ± 4.5	9.4 ± 2.0
		*P*	NS	NS	NS	NS
pH		Before	5.90 ± 0.05	5.64 ± 0.15	5.83 ± 0.10	5.82 ± 0.13
		After	5.83 ± 0.15	5.48 ± 0.07	5.81 ± 0.02	5.78 ± 0.07
		*P*	NS	NS	NS	NS
Microbial population	Total bacteria, × 10^11^/g	Before	1.51 ± 1.12	9.00 ± 7.09	13.01 ± 6.65	0.90 ± 0.55
		After	3.04 ± 1.07	7.52 ± 5.96	9.50 ± 5.90	2.48 ± 0.54
		*P*	NS	NS	NS	NS
	Total archaea, × 10^8^/g	Before	8.04 ± 1.01	4.84 ± 0.74	5.85 ± 1.50	7.05 ± 1.94
		After	17.38 ± 6.16	15.08 ± 3.21	12.79 ± 2.08	16.04 ± 2.92
		*P*	NS	**	*	*

*NS* non-significant

^†^0.05 ≤ *P* < 0.1

*0.01 ≤ *P* < 0.05

**P < 0.01

### Microbial community dynamics in control animals

**Fig. 1:**
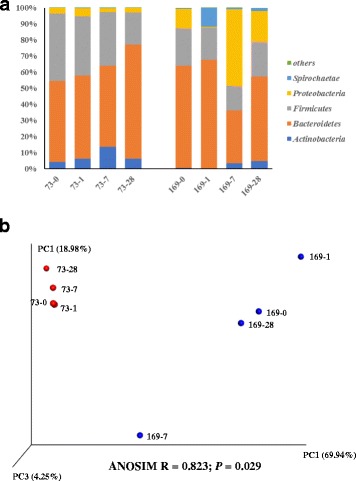
Microbial community dynamics in control animals. **a** Bacterial phyla identified along the experiment. **b** PCoA plot of the bacterial profiles from 454 sequencing

The bacterial profiles of the control animals were firstly compared to examine whether changes occurred in animals without being subjected to transfaunation. A total of 95,930 sequences (11,991 ± 3270 seqs/sample) were obtained from the control animals and assigned to 744 unique OTUs (221 ± 53 OTUs/sample). Although the two control animals were maintained under identical conditions throughout the experiment period, the proportion of *Bacteroidetes* increased and the proportion of *Fircimutes* decreased at D28 for Animal 73 and the proportion of *Spirochaetes* was higher at D1 and the proportion of *Proteobacteria* spiked at D7 while the communities of other time points were similar (Fig. 1a) for Animal 169. The bacterial communities differed between the two animals (Fig. 1b).

### Identification of rumen microbiome using pyrosequencing

In-depth sequence analysis was applied to examine the bacterial profiles in the rumen collected at D0, D1, D7, and D28. In total, 789,175 sequences passed quality filter (12,330 ± 4124 seqs/sample) and were assigned to 2243 unique OTUs (242 ± 93 OTUs/sample), belonging to 15 bacteria phyla, 64 families, and 99 genera. The alpha diversity indices of each sample are listed in Additional file 1: Table S2. Most of the samples harbored Bacteroidetes and *Firmicutes* as the most abundant phyla, with only three exceptions (D1 samples of Animal 59, Animal 67, and Animal 247) containing highly abundant *Actinobacteria* (81, 51, and 43%, respectively).

### Distinction of microbial community before rumen contents exchange

**Fig. 2:**
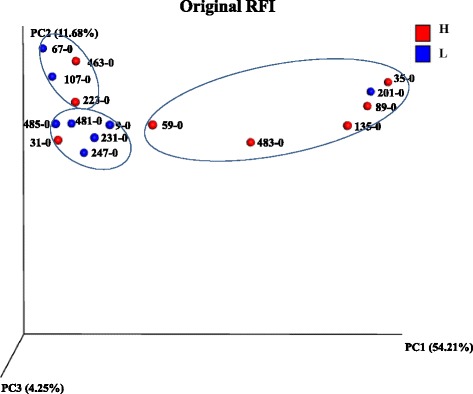
PCoA plot of bacterial profiles of D0 from 454 pyrosequencing

The microbial profiles of D0 were firstly compared to identify whether there was distinction of the microbiota prior to the transfaunation. PCoA analyses showed that only very few clusters were formed according to different grouping criteria. As shown in Fig. 2, three clusters were formed based on animal original RFI, where four H-RFI animals formed one cluster, five L-RFI animals formed another cluster, while two H-RFI and two L-RFI animals formed the third cluster. In addition, two animals (Animals 201 and 31) hosted bacterial communities closer to the animals belong to the opposite RFI group rather than to the animals belong to the same RFI group.

### Host-specific rumen microbial adaptation after rumen transplantation

**Fig. 3:**
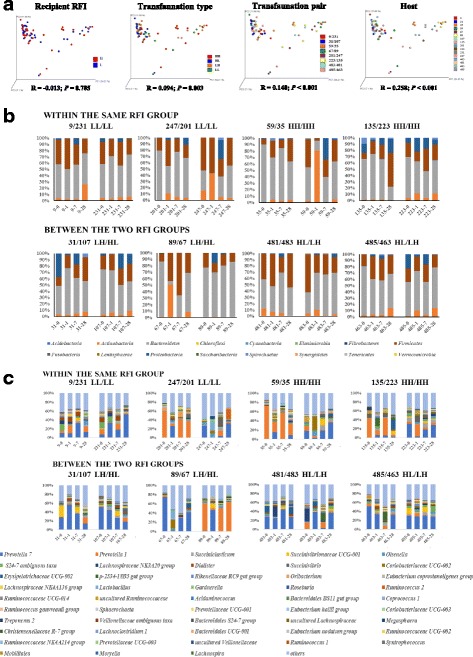
Recovery patterns of the bacterial communities. **a** Clustering of bacterial profiles at D0, D1, D7, and D28 with different classifications. **b** Bacterial community alteration along experiment at phylum level. **c** Bacterial diversity alteration along experiment at genus level

**Fig. 4:**
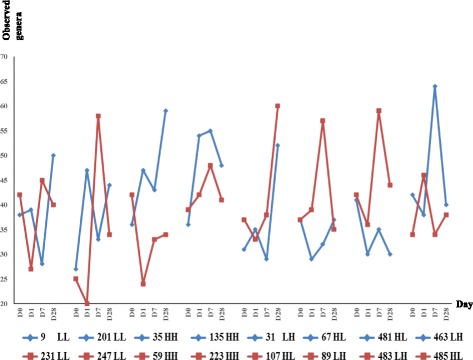
Changes in the observed OTUs along the re-establishment process

The factors impacting the clustering of the overall microbial profiles after rumen transfer were analyzed. RFI did not affect the clustering; the microbial profiles slightly differed among the four transfaunation types, the transfaunation pair, and the host as indicated by the low ANOSIM R values although *P* value all indicated significance (Fig. 3a). The microbial phylotype fluctuation was then analyzed at phyla (Fig. 3b) and genus (Fig. 3c) level, respectively. The changes of the microbial profiles after transfaunation were unique for each exchange pair and each individual animal, and the microbial diversity altered with distinctive patterns for each animal regardless of the transfaunation type and transfaunation pair. Soon after the exchange procedure, the D1 profiles of Animals 31/481/483 were similar to the donors’ D0 profiles, the D1 profiles of Animals 9/107 remained similar as their own D0 profiles, while the D1 profiles of the rest of all animals was neither similar to the D0 profiles of the donor nor to their own D1 profiles (Fig. 3b, c). As the adaptation progressed, the microbial compositions altered dramatically for most of the animals and did not resemble to that of the donor. The only exception was for Animals 89 and 485, who had their D28 profiles similar to the D0 profiles of the donor (Fig. 3b, c). Similarly, the bacterial community diversity of each animal also fluctuated (Fig. 4): eight animals had less phylotypes (e.g., Animal 231 D0 vs. D1: 42 vs. 27) while eight animals (e.g., Animal 201 D0 vs. D1: 27 vs. 47) has more phylotypes being identified at D1 soon after the transfaunation procedure. Comparable host-specific change patterns were also observed with UPGMA clustering analyses for both 454 pyrosequencing data and fingerprinting profiling data (Additional file 2: Figures S1-S3).

### Different responses of each microbial phylotype to the transfaunation process

**Fig. 5:**
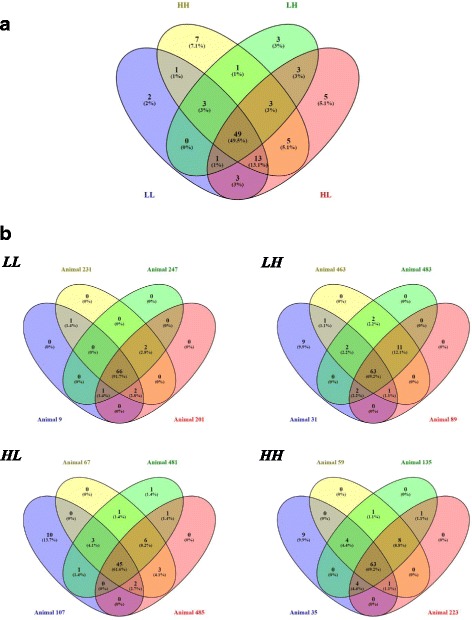
Common and distinctive bacterial genera among the samples by Venn’s diagram. **a** Genera distribution among the four exchange types. **b** Genera distribution among individuals belonging to the same transfaunation type

The distribution of each identified bacterial genus differed among the four transfaunation types (Fig. 5a), and variation in bacterial genus distribution was further observed for individuals belonging to the same transfaunation type (Fig. 5b). The relative abundance of the observed bacterial genera was then analyzed for each individual animal. Only the genus displaying at least twofold difference in relative abundance between D0 and D28 samples were determined as “decreased” or “increased” genus. The changing trend of each genus for the animals within the same pair were not necessarily the same: some genera showed the same changing trend for both hosts (e.g., in Animal pair 9/231, *Succiniclasticum* increased in both animals: 1.3 to 4.6% and 1.6 to 3.3%, respectively), and some genera showed different changing patterns for the two hosts (e.g., in Animal pair 201/247, *Coriobacteriaceae UCG-002* increased in Animal 201 from < 0.01 to 0.6% and decreased in Animal 247 from 2.6 to 0.04%).

As shown in Additional file 1: Table S3, even for the genera belonging to the same changing trend (either “increased” or “decreased”), their changing process through the experimental period was different for individuals. The genera which had the same changing trend after transfaunation in at least three animals of the same transfanation type (LL/HH/LH/HL) were plotted in Additional file 2: Figure S4. Some genera such as *Lachnospira* in Animal 59 (HH) and in Animal 223 (HH) (Additional file 2: Figure S4) gradually increased with days, while some genera such as *Gardnerella* in Animal 35 (HL) and Animal 463 (Additional file 2: Figure S4) quickly increased significantly after transfaunation (D1 vs. D0) and fluctuated during the adaptation period (D1–D28). Some genera had opposite responses in animals belonging to different exchange types, and some genera even had opposite response in animals belonging to the same exchange type (Additional file 1: Table S3).

The speed of microbial adaptation differed for individual microbial phylotype. While in some cases that the relative abundance of the bacterial genera increased/decreased gradually and only had their abundance significantly different at D28 (e.g., *Syntrophococcus* in Animal 9, Additional file 2: Figure S4), other genera altered soon after exotic microbiome was introduced (e.g., *Lachnospira* in Animal 59, Additional file 2: Figure S4). The alteration speed for the same bacterial genus was also different in different hosts: for example, *Lachnospira* was only significantly higher at D28 compared to D0 in Anima 223 but was more abundant from D1–D28 in Animal 59 (Additional file 2: Figure S4).

### Phylotype of interest for microbial manipulation from RFI aspect

To evaluate the microbial manipulation potentials, exchanges between animals of different RFI classes were further analyzed. While most of the genera showed specific changing trends for each individual animal, only three genera displayed the same changing trend in three out of four animals belonging to the same transfaunation type, which the RFI classes of the two animals differed (Additional file 2: Figure S4): In LH group, *Lactobacillus* and *Coprococcus 1* increased in Animals 31/483/463, and *Coriobacteriaceae UCG-003* increased in Animals 31/89/463.

### Co-variation of microbiota and feed efficiency in response to rumen contents exchange

To further explore the effect of rumen exchange on feed efficiency, the co-variation of microbial profiles and feed efficiency before and after exchange were performed. As shown in Additional file 1: Table S4, the microbial profiles and FCR of six animals (Animals 9, 231, 107, 463, 485, and 223) remained similar before and after rumen transfaunation; while both the microbial profiles and FCR altered significantly in two animals (Animals 247 and 135). In the meantime, the microbial profiles changed while FCR remained stable in five animals (Animals 201, 67, 89, 483, and 59), and FCR changed dramatically with the microbial profiles remained similar in three animals (Animals 31, 35, and 481).

### Correlation between microbial phylotypes and rumen fermentation parameters

As shown in Additional file 1: Table S5, the predominant genera *Prevotella 1* was associated with isovalerate molar portion and mean pH; two of the major phylotye (relative abundance > 0.1) *Erysipelotrichaceae UCG-002* and *Coriobacteriaceae UCG-002* were associated with valerate molar portion; one of the major phylotype *Lachnospiraceae NK4A136* group was associated with propionate molar portion. Association was also observed for minor genera (relative abundance < 0.1) and the molar portion of individual VFAs, NH_3_-N, and rumen pH.

## Discussion

The success of fecal microbial transplantation in mice which redirected symbiotic microbiota and thereafter improved animal health status [4–7] has shed light on redirection of gut microbiota. Similarly, the re-establishing and maintaining a healthy and efficient rumen microbiota through rumen microbial transplantation could potentially enhance rumen function. However, most of the previous studies employing the “whole rumen content exchange” method focused on the changes in host behavior, metabolism, and product quality measurements [30–32]. Currently, only one study examined the bacterial community in the dairy cows subjected to rumen content cross-inoculation, where individual-dependent rumen microbiota re-establishment succession was reported, with microbiome more likely resumed its original composition gradually [9]. However, in that preliminary study, only four dairy cows were tested and the microbial profiles were performed using low-resolution method (fingerprinting), and the rinse step was not employed to ensure the complete removal of rumen content. Further, only rumen pH and total VFA were measured as animal performance indicators in the dairy study, and the association between host performance and microbial phylotypes has not been reported.

In our current study, 16 animals were involved which has increased the statistical power to cope with the limitations in lower number of animals by Weimer et al. [9]. In addition, the minimum three-times rinses applied in the current study assured effective removal of the rumen content. In this step, shorten the processing time was essential to limit the adverse effects to the hosts, such as the uncomfortableness of the animal and the potential damage to the rumen wall. We managed to minimize the cross-over effects by the original rumen microbiota, although the epimural microbiota was unable to be completely depleted by such rinse step. It is possible to use the antibiotic spray to treat the rumen after rinse steps, which may help remove the tissue-attached microbiota if this community has an effect on rumen microbial re-establishment. However, it is unknown whether the remaining antibiotics would affect the transplanted microbiome and/or antibiotics can act instantaneously and effectively remove the epithelial attached microbes, and thus further influence the microbial functions after the transfaunation procedure. Therefore, we only focused on removing and exchanging the rumen content microbiota. Since the content microbiota is the main fermenter in the rumen [33] and accounts for up to 95% of total population, the current study is valuable to understand the microbial changes after introducing exogenous microbiota. Besides, the variation in microbial profiles observed for each individual animal prior to the exchange (Fig. 2) was ideal for transplantation in that the more distinctive the donor microbiota is, the more differences it may introduce to the recipient.

In all of the 16 animals with transfaunation conducted, individual variation in the microbial profiles was seen for each animal (Fig. 3). The unique clustering patterns of the microbial profiles from both pyrosequencing and PCR-DGGE further supported the divergence among individuals (Additional file 2: Figures S1-S3), although discrepancies on the UPGMA clustering were observed owing to the detection resolution differences of the two methods (Additional file 2: Figures S1-S3). This host specific microbiome re-establishment may be due to the variance of completeness in removing the rumen content microbiota, although we used identical procedures to each animal. The remaining microbiota (such as epithelial attached microbes), although very limited, may still affect the re-establishment of the rumen microbiome with the content transfaunation. Another factor leading to the inconsistent responses to transfaunation may be the individual variance of the host animals. It was reported that in the human fecal transplantation, microbiome from the single donor had developed into distinguishable microbiome in the four recipient patients [8]. Our results implicate the importance of host variation, which may also contribute to the unique microbial adaptation patterns as well as the dynamics of the symbiotic microbiome for individual animals. As reported previously, the different genetic backgrounds of host animal also play a role in affecting its rumen microbiota [18]. The host-specific re-establishment process observed in the current study was comparable with a previous report, where the rumen microbiota of each individual animal adapted to the environmental changes in a unique way [34]. However, as no parentage test has been done in the current study, we were not able to conduct further analyses to attribute such discrepancy with respect to host genetic variation.

Besides the community structural variation (Fig. 3) being observed for individual animals, the extent and speed of each microbe varied after the exchange process indicates that the unique physiological and biochemical features of each microbial phylotype may also contribute to the different adaptation patterns. As each animal may have variations in ruminal parameters such as pH, volume, temperature, passage rates, etc., which affects its symbiosis with different microbial species, the microbes responded to the environmental changes in different ways (Additional file 1: Table S4 and Additional file 2: Figure S4). The rumen microbial species were anaerobes [35], therefore it was hypothesized that bacteria species richness would decrease soon after the transfaunation. Surprisingly, species richness of some recipient animals remained similar (e.g., Animal 231) or even became significantly higher (e.g., Animals 201 and 35) after receiving the content from donor animals. The species richness at D1 of the recipients was not necessarily associated with that of the donor (e.g., Pairs 201/247 and 483/481) (Fig. 4). In addition, none of the bacterial communities among the animals subjected to transfaunation retrieved an identical structure as those prior to the experiment (Additional file 2: Figures S1-S3). This suggests that some of the phylotypes may be eliminated permanently while some of the phylotypes were only affected temporarily. Further studies examining the metagenomes may explain the differed responses of individual microbial phylotypes upon transfaunation.

One of the main concerns about introducing exotic microbiota to the rumen was whether the normal rumen function can be restored or not. As shown in Table 2, most of the fermentation parameters remained stable before and after the exchange process, indicating that the exchange procedures in the current study were completed without interfering normal rumen functions. The unchanged fermentation parameters can be explained by the relative stable microbial communities before and after the experiment procedures that the bacteria population did not change (Table 2), and only a few of the identified bacterial genera either increased or decreased while the majority remained similar for each animal (Additional file 1: Table S4).

Among all of the bacterial genera, the microbial alterations in animals receiving rumen content from the donors with identical RFI class (animals of HH and LL group) showed individuality, indicating that host genetic variation may play important roles determining the symbiotic microbiome. Rather, the phylotype changes showed similar trends in animals obtaining rumen content from the opposite RFI class (animals of HL and LH group) may be of more importance for discussion in terms of their implication on impacting recipient feed efficiency. *Lactobacillus*, which increased in LH animals, was shown to play an important role in rumen acidosis [36]. Although the actual roles of *Lactobacillus* in influencing steer RFI ranking is still under exploration, higher *Lactobacillus* was reported to be associated with efficient hosts in monogastric animals including pigs [37] and chicken [38]. It can be speculated that the efficient hosts (L-RFI) may have preference to host higher abundant *Lactobacillus*, and thus restoring its fermentation capacity. *Coriobacteriaceae* and *Coprococcus* were both had the greatest abundance in low-intake-high-gain efficient steers [39]. The increased population of *Coriobacteriaceae UCG-003* and *Coprococcus 1* in LH steers after transfaunation may suggest a similar mechanism as that for *Lactobacillus*, that the L-RFI steers would trigger certain mechanisms to promote these key feed efficiency-related bacteria after being challenged by transfaunation procedures, so that to maintain the fermentation efficiency. In addition, while common changing trends of these three phylotypes were observed from animals of the same transfaunation types, they may serve as the target of rumen manipulation.

The association between the microbial phylotypes and the rumen fermentation parameters identified in the current study may provide additional information to understand better how the entire microbial community adjusts its structure to maintain its proper functions after transfaunation (Additional file 1: Table S5). However, none the three bacterial phylotypes with similar changing trend in LH animals (*Lactobacillus*, *Coriobacteriaceae UCG-003*, and *Coprococcus 1*) were associated with any measured parameters and thus may explain why the fermentation parameters remained similar after transfaunation.

The main limitation for the current study was that the animals were not subjected to RFI evaluation after transfaunation due to the limited resources and time to measure this trait. However, we did evaluate FCR after rumen contents exchange. It was reported that FCR (feed/gain) is moderately to highly correlated with RFI (*R* = 0.45–0.85) [19, 40] and thus, we considered FCR can be used as an indirect indicator for RFI and they both represent the feed efficiency. Further, rumen VFA/NH_3_-N which indicate cattle rumen fermentation, could partially contribute to the variation of host feed efficiency [14, 41, 42]. It is known that VFA measured in the rumen are results of microbial production and host absorption. Although the correlation of FCR before and after transfaunation was not observed (Additional file 1: Table S1), it is noticeable that synchronized changes in both FCR and microbial profiles before and after the transfaunation only occurred in two animals (Animals 247 and 135, Additional file 1: Table S4). This co-variation suggests that rumen microbial structure in these two animals could be associated with host feed efficiency, and such changes are specific for individual animals rather than exchange pair/exchange type dependent. For the five animals (Animals 201, 67, 89, 483, and 59) with changed microbiome and stable FCR, the introduced microbiome may successfully compensate the function of the innate microbiome and adapt to the host rumen environment well, to allow the animals to have similar performance. While for the three animals with similar microbiome but changed FCR (Animals 31, 481, and 35), it can be speculated that these two animals may require longer time to allow its rumen microbiome to maintain its normal function after undergoing the transfaunation process. These results further emphasize the individual variation, that host factors should be considered in the future practices on rumen microbial manipulation. Future analyses combining host genetics, individual physiological traits, fermentation parameters, and microbial ecology should be more comprehensive in evaluating the transfaunation process, and the follow-up RFI measurement will thus further validate the improvement of animal performance, if there is any.

Another drawback of this study is that we could not evaluate the effect of taking out rumen content and returning it on the microbial profile changes. However, no significant difference in total bacterial population on D1 suggests that the bacteria were not affected by this process at population level. To validate the host microbial dynamics after receiving exotic rumen content, the control animals were intended to set without having their rumen content being removed, instead of having their content taken out and put back. In theory, the microbial profiles of the two untouched control animals were expected to be unchanged or at least more stable than the animals subjected to transfaunation. The similarities of microbial profiles and the identified phylotypes were indeed higher within control animals compared to that of the animals with rumen content exchanged (Fig. 1). However, changes in both bacterial and archaeal communities along the experiment were still observed for the control animals, particularly the sudden spike of *Proteobacteria* for Animal 169 at D7 (Fig. 1a). As no take-out/re-insert action was conducted on the control animals, the fluctuation of the relative abundance of each identified phyla proved that the rumen microbiota is dynamic. Therefore, with the “innate dynamics” existing, even “remove-return” control animals are included, it is still impossible to distinguish whether the microbial community changes are from ‘innate dynamics’ or from the content ‘remove-return’ process. To perfectly address such limitations, “germ-free” ruminant models are needed to receive microbiome from efficient and inefficient animals, respectively. The animal performance being measured from both recipients and donors can provide better explanation whether the recipient animal has adopted “donor performance” through the incoming microbiome. The original experimental design also included two L-RFI animals as control, however, as both of them were later found not properly castrated and had difficulties in adapting to the transfaunation procedure, they were removed from the study. Thus, the microbial dynamics observed in the current study may be biased by the H-RFI steers.

It is also noticeable for the unique dynamics of particular bacterial genera among the treated animals, such as the predominance of *Prevotella* for Animal 35 at D0 which occupied approximately 90% of the bacterial communities and the high proportion of *Shapea* for Animal 135 at D28 (Additional file 2: Figure S4). Additionally, the high proportion of *Actinobacteria* in D1 samples of Animal 59 (78%) and Animal 67 (64%) (Fig. 3b) suggested that the sudden increase of these non-predominant phylotypes might not necessarily be associated with the nature of the donor microbiota. Rather, this may be associated with the transfaunation procedure that such process affected the microbiome which was transferred to these two animals significantly. It would also be possible that host-microbiome mutual adaptation play an important role leading to this result that these two animals showed higher repellence of other phylotypes while accepting non-native rumen content. Although we were unable to explain the high proportion of these bacterial genera at a certain time point of the microbial re-establishing process, the comparable dynamics in microbiome was also reported in the human fecal microbiome transplantation experiment [8]. In future study, it is necessary to involve multiple samples prior to the transfaunation process, so as to compare the microbial dynamics prior and after receiving non-native microbiota, and to understand the mechanisms how microbial homeostasis is achieved.

## Conclusions

In conclusion, the transition of bacteria and archaea communities, as well as the adaptation speed and extent of each identified microbial phylotype was specific for individual host. The bacterial and archaeal community re-establishment of the same host was not necessarily correlated, suggesting a more complex host-related regulatory system in its symbiotic microbiota development. Among all of the bacterial phylotypes, *Lactobacillus*, *Coriobacteriaceae*, and *Coprococcus*, may have higher manipulation potential compared to other genera by means of content transfaunation, while other methods should be developed to regulate the 18 microbial phylotypes who are associated with rumen VFA and NH_3_-N parameters. Although the trend of microbial profile changes were associated with cattle FCR alteration in the majority of the animals examined, further analyses combining host individual genetic and physiological traits together with microbial data will be more complete in understanding the entire microbial re-establishment. Although the relationship between re-establishment of rumen microbiome and RFI outcome is not conclusive, a study including over 700 steers that also uses RFI as an indicator for host performance is in progress in order to identify the correlations between host genetic markers and each symbiotic microbial species. By clarifying how host genetics influence its symbiotic microbiota, we may be able to explain the individualized microbial adaptation patterns of the current study better. Future functional analyses on such process may further facilitate in rumen microbial manipulation.

## Additional files

Additional file 1: Supplementary Materials and Tables: Zhou et al. Microbiome rr supplementary materials and tables. (DOCX 47 kb)

Additional file 2: **Figure S1.** UPGMA clustering of the bacterial profiles by 454 pyrosequencing. **Figure S2.** UPGMA clustering of the long-term bacterial and archaeal profile changes by PCR-DGGE. **Figure S3.** UPGMA clustering of the short-term bacterial and archaeal profile changes by PCR-DGGE. **Figure S4.** The bacterial genera with relative abundance increased after transfaunation. The relative abundance was presented as proportions. The phylotypes were either increased after transfaunation. (ZIP 352 kb)
