# Supplementary material for: Assessment of microbiome changes after rumen transfaunation: implications on improving feed efficiency in beef cattle
Source: Microbiome. 2018 Mar 27;6:62. doi: 10.1186/s40168-018-0447-y (PMC5869788; doi:10.1186/s40168-018-0447-y)
Supplement: Supplementary file 1 — Supplementary Materials and Tables: Zhou et al. Microbiome rr supplementary materials and tables. (DOCX 47 kb) [file 40168_2018_447_MOESM1_ESM.docx]

**Microbiome**

Assessment of microbiome changes after rumen transfaunation: implications on improving feed efficiency in beef cattle

Mi Zhou^1^, Yong-Jia Peng^2^, Yanhong Chen^1^, Christen M. Klinger^1^, Masahito Oba^1^, Jian-Xin Liu^2^ and Le Luo Guan^1*^

^1^ Department of Agricultural, Food and Nutritional Science, University of Alberta, Edmonton, Alberta, Canada, T6G 2P5; ^2^ College of Animal Sciences, Zhejiang University, Hangzhou, 310058, Zhejiang, China

*Corresponding Author:

Dr. Leluo Guan, 416F Agr/For Centre, University of Alberta, Edmonton, Alberta, Canada, T6G 2P5. Tel: 1-780-4922480; Fax: 1-780-4924265. E-mail: [lguan@ualberta.ca](mailto:lguan@ualberta.ca)

**Supplementary Materials**

**Transfaunation procedure:**

As the rumen microbiome are sensitive to oxygen exposure, and long-time exposure of rumen content to the air may lead to reduction of microbial activities and/or depletion of strict anaerobes, it was necessary to complete the transfaunation procedure as soon as possible. On the day of transfaunation, the PBS buffer was pre-warmed on site prior to the experiment procedures. Once the preparation was completed, four people worked together to handle one animal a time. We emptied all contents within 5 min (1 person) and then we washed the rumen with PBS buffer three times which was done within 5 min (1 person), and we put back the contents within 5 min (1 person). And one person was assigned to provide buckets, change buffer and dump buffer and so on. The entire procedure for each transfaunation pair was completed within 15 min.

QIIME scripts for the current study:

*Step 1: split library*

split_libraries.py -m mappingfile/ID.txt -f seqs/ID.fna -q qual/ID.qual -o Filtered_reads/ID -l 100 -L 1000 -s 20 -M 0 -b 5 -z truncate_only

*Step 2: OTU picking*

pick_closed_reference_otus.py -i seqs.fna -r 97_otus_16S.fasta -t consensus_taxonomy_7_levels.txt -o closed_otus -p parameter.txt

*Step 3: Alpha diversity*

alpha_rarefaction.py -i closed_otus/otu_table.biom -o alpha_rarefaction -m map.txt -p alpha_params.txt -t 97_otus.tre

*Step 4: Beta diversity*

beta_diversity_through_plots.py -i closed_otus/otu_table.biom -o beta_diversity -t 97_otus.tre -m map.txt -e ‘lowest OTU number’

*Step 5: Jackknifed beta diversity*

jackknifed_beta_diversity.py -i closed_otus/otu_table.biom -o jbeta_diversity -t 97_otus.tre -m map.txt -e ‘75% of the lowest OTU number’

*Step 6: ANOSIM*

compare_categories.py --method anosim -i weighted_unifrac_dm.txt -m map.txt -c host -o anosim_out_host -n 50000

**Supplementary Tables**

**Table S1.** Correlation of fermentation parameters before and after transfaunation.

| Parameters | R | *P* |
| --- | --- | --- |
| DMI | 0.500 | 0.05 |
| ADG | -0.080 | 0.77 |
| FCR | -0.292 | 0.27 |
| pH | 0.410 | 0.11 |
| NH3-N | 0.422 | 0.10 |
| Total VFA | 0.604 | 0.01 |
| Acetate | 0.364 | 0.17 |
| Propionate | 0.417 | 0.11 |
| Butyrate | 0.407 | 0.12 |

**Table S2.** Summary of alpha diversity indices of each sample.

| Sample | OTUs | Chao1 | Good’s coverage | Shannon | Simpson |
| --- | --- | --- | --- | --- | --- |
| 9-0 | 15024 | 313 | 0.994 | 4.509 | 0.903 |
| 9-1 | 10666 | 376 | 0.991 | 4.997 | 0.920 |
| 9-7 | 11918 | 272 | 0.994 | 3.943 | 0.877 |
| 9-28 | 10351 | 428 | 0.989 | 5.533 | 0.954 |
| 31-0 | 18126 | 196 | 0.996 | 3.023 | 0.765 |
| 31-1 | 12222 | 254 | 0.995 | 3.015 | 0.699 |
| 31-7 | 7590 | 184 | 0.995 | 3.518 | 0.814 |
| 31-28 | 10224 | 443 | 0.982 | 4.758 | 0.907 |
| 35-0 | 13443 | 439 | 0.989 | 2.886 | 0.608 |
| 35-1 | 7581 | 444 | 0.983 | 5.320 | 0.952 |
| 35-7 | 9557 | 632 | 0.982 | 4.534 | 0.855 |
| 35-28 | 6752 | 573 | 0.974 | 6.382 | 0.972 |
| 59-0 | 11694 | 458 | 0.990 | 4.522 | 0.892 |
| 59-1 | 15238 | 195 | 0.995 | 2.711 | 0.659 |
| 59-7 | 14352 | 280 | 0.994 | 3.977 | 0.889 |
| 59-28 | 8041 | 261 | 0.994 | 3.546 | 0.746 |
| 67-0 | 19179 | 314 | 0.993 | 2.729 | 0.697 |
| 67-1 | 9844 | 297 | 0.992 | 3.572 | 0.830 |
| 67-7 | 11406 | 165 | 0.997 | 2.307 | 0.631 |
| 67-28 | 17767 | 252 | 0.995 | 3.500 | 0.801 |
| 89-0 | 5693 | 637 | 0.985 | 4.621 | 0.896 |
| 89-1 | 10872 | 367 | 0.990 | 3.774 | 0.840 |
| 89-7 | 10375 | 602 | 0.964 | 6.006 | 0.955 |
| 89-28 | 10262 | 652 | 0.981 | 4.839 | 0.883 |
| 107-0 | 18095 | 206 | 0.996 | 3.337 | 0.753 |
| 107-1 | 11454 | 154 | 0.997 | 3.454 | 0.801 |
| 107-7 | 12050 | 401 | 0.993 | 4.167 | 0.885 |
| 107-28 | 7293 | 488 | 0.983 | 5.446 | 0.949 |
| 135-0 | 23741 | 399 | 0.991 | 3.833 | 0.796 |
| 135-1 | 15326 | 542 | 0.989 | 4.904 | 0.882 |
| 135-7 | 8650 | 631 | 0.981 | 6.102 | 0.967 |
| 135-28 | 10747 | 442 | 0.989 | 4.479 | 0.862 |
| 201-0 | 23773 | 317 | 0.993 | 3.366 | 0.772 |
| 201-1 | 12788 | 344 | 0.993 | 4.416 | 0.890 |
| 201-7 | 13717 | 379 | 0.992 | 3.857 | 0.828 |
| 201-28 | 12138 | 544 | 0.988 | 5.322 | 0.929 |
| 223-0 | 18882 | 404 | 0.991 | 4.079 | 0.822 |
| 223-1 | 16179 | 476 | 0.990 | 4.669 | 0.892 |
| 223-7 | 11248 | 428 | 0.990 | 5.065 | 0.941 |
| 223-28 | 7304 | 224 | 0.992 | 4.547 | 0.897 |
| 231-0 | 18535 | 411 | 0.992 | 4.678 | 0.916 |
| 231-1 | 12442 | 179 | 0.997 | 3.320 | 0.784 |
| 231-7 | 12137 | 526 | 0.988 | 5.303 | 0.946 |
| 231-28 | 9555 | 289 | 0.993 | 3.094 | 0.654 |
| 247-0 | 7101 | 130 | 0.995 | 2.857 | 0.755 |
| 247-1 | 23395 | 172 | 0.997 | 3.644 | 0.890 |
| 247-7 | 13753 | 558 | 0.988 | 5.730 | 0.950 |
| 247-28 | 8913 | 244 | 0.992 | 2.996 | 0.726 |
| 463-0 | 10061 | 337 | 0.990 | 4.048 | 0.859 |
| 463-1 | 8974 | 225 | 0.993 | 4.353 | 0.899 |
| 463-7 | 10721 | 714 | 0.983 | 5.132 | 0.877 |
| 463-28 | 8761 | 337 | 0.990 | 4.250 | 0.838 |
| 481-0 | 15770 | 223 | 0.996 | 3.945 | 0.870 |
| 481-1 | 10256 | 200 | 0.995 | 3.490 | 0.822 |
| 481-7 | 17094 | 233 | 0.995 | 3.301 | 0.763 |
| 481-28 | 11994 | 244 | 0.995 | 3.536 | 0.857 |
| 483-0 | 15138 | 462 | 0.990 | 4.174 | 0.807 |
| 483-1 | 10209 | 202 | 0.995 | 3.556 | 0.825 |
| 483-7 | 5430 | 602 | 0.966 | 5.709 | 0.909 |
| 483-28 | 13526 | 343 | 0.993 | 3.976 | 0.805 |
| 485-0 | 11563 | 241 | 0.995 | 3.828 | 0.862 |
| 485-1 | 10633 | 452 | 0.990 | 5.012 | 0.913 |
| 485-7 | 10435 | 269 | 0.994 | 3.654 | 0.803 |
| 485-28 | 11217 | 353 | 0.992 | 3.831 | 0.852 |
| 73-0 | 18479 | 248 | 0.995 | 4.230 | 0.897 |
| 73-1 | 13008 | 354 | 0.993 | 4.527 | 0.913 |
| 73-7 | 10291 | 356 | 0.992 | 4.840 | 0.924 |
| 73-28 | 11456 | 244 | 0.995 | 3.273 | 0.737 |
| 169-0 | 11378 | 409 | 0.990 | 4.528 | 0.909 |
| 169-1 | 8328 | 413 | 0.989 | 3.870 | 0.830 |
| 169-7 | 15027 | 272 | 0.994 | 3.262 | 0.743 |
| 169-28 | 7963 | 490 | 0.982 | 5.143 | 0.920 |

**Table S3.** Genera that were either increased or decreased after transfaunation.

| Transfaunation type | Animal | Genus | D0 | D1 | D7 | D28 |
| --- | --- | --- | --- | --- | --- | --- |
| LL | 9 | *S24-7 ambiguous taxa* | 0.2238 | 0.0078 | 0.0372 | 0.0051 |
|  |  | *Acidaminococcus* | 0.0154 | 0.0130 | 0.0065 | 0.0028 |
|  |  | *Sphaerochaeta* | 0.0000 | 0.0084 | 0.0007 | 0.0085 |
|  |  | *Lachnoclostridium 1* | 0.0013 | 0.0009 | 0.0003 | 0.0002 |
|  | 231 | *Eubacterium hallii group* | 0.0106 | 0.0033 | 0.0022 | 0.0035 |
|  |  | *Veillonellaceae ambiguous taxa* | 0.0171 | 0.0026 | 0.0012 | 0.0001 |
|  | 201 | *Prevotella 7* | 0.0003 | 0.3437 | 0.0009 | 0.0021 |
|  |  | *Succiniclasticum* | 0.0003 | 0.0525 | 0.0010 | 0.0180 |
|  |  | *Olsenella* | 0.0007 | 0.0497 | 0.0147 | 0.0207 |
|  |  | *S24-7 ambiguous taxa* | 0.0015 | 0.0150 | 0.0052 | 0.0264 |
|  |  | *Succinivibrio* | 0.0000 | 0.0289 | 0.0061 | 0.1106 |
|  |  | *Coriobacteriaceae UCG-002* | 0.0000 | 0.0401 | 0.0164 | 0.0055 |
|  |  | *p-2534-18B5 gut group* | 0.0032 | 0.0001 | 0.0006 | 0.0000 |
|  |  | *Lachnospiraceae NK4A136 group* | 0.0000 | 0.0188 | 0.0001 | 0.0031 |
|  |  | *Gardnerella* | 0.0000 | 0.0153 | 0.0040 | 0.0102 |
|  |  | *Roseburia* | 0.0003 | 0.0025 | 0.0005 | 0.0021 |
|  |  | *Eubacterium hallii group* | 0.0000 | 0.0065 | 0.0001 | 0.0005 |
|  |  | *uncultured Lachnospiraceae* | 0.0001 | 0.0009 | 0.0005 | 0.0019 |
|  |  | *Eubacterium nodatum group* | 0.0000 | 0.0012 | 0.0004 | 0.0003 |
|  |  | *Ruminococcaceae UCG-002* | 0.0001 | 0.0008 | 0.0004 | 0.0003 |
|  |  | *Syntrophococcus* | 0.0001 | 0.0033 | 0.0004 | 0.0019 |
|  |  | *Mobilitalea* | 0.0000 | 0.0009 | 0.0001 | 0.0003 |
|  |  | *Lachnospira* | 0.0000 | 0.0014 | 0.0002 | 0.0021 |
|  | 247 | *Rikenellaceae RC9 gut group* | 0.0001 | 0.0003 | 0.0126 | 0.0013 |
|  |  | *uncultured Ruminococcaceae* | 0.0000 | 0.0001 | 0.0009 | 0.0151 |
|  |  | *Sphaerochaeta* | 0.0000 | 0.0000 | 0.0161 | 0.0016 |
|  |  | *Prevotellaceae UCG-001* | 0.0000 | 0.0007 | 0.0006 | 0.0001 |
|  |  | *Coriobacteriaceae UCG-003* | 0.0018 | 0.0053 | 0.0039 | 0.0062 |
|  |  | *Bacteroidales S24-7 group* | 0.0000 | 0.0001 | 0.0012 | 0.0001 |
|  |  | *Lachnoclostridium 1* | 0.0000 | 0.0001 | 0.0115 | 0.0002 |
|  |  | *Eubacterium nodatum group* | 0.0000 | 0.0000 | 0.0055 | 0.0011 |
|  |  | *uncultured Veillonellaceae* | 0.0003 | 0.0008 | 0.0020 | 0.0008 |
|  |  |  |  |  |  |  |
| HH | 35 | *Prevotella 7* | 0.0005 | 0.2000 | 0.0065 | 0.1263 |
|  |  | *Succiniclasticum* | 0.0002 | 0.0024 | 0.0055 | 0.0179 |
|  |  | *Olsenella* | 0.0002 | 0.0135 | 0.0060 | 0.0087 |
|  |  | *S24-7 ambiguous taxa* | 0.0013 | 0.0146 | 0.0059 | 0.0129 |
|  |  | *Coriobacteriaceae UCG-002* | 0.0003 | 0.0273 | 0.0071 | 0.0025 |
|  |  | *Rikenellaceae RC9 gut group* | 0.0124 | 0.0187 | 0.0267 | 0.0281 |
|  |  | *Gardnerella* | 0.0000 | 0.0021 | 0.0028 | 0.0059 |
|  |  | *Sphaerochaeta* | 0.0000 | 0.0355 | 0.0006 | 0.0021 |
|  |  | *Veillonellaceae ambiguous taxa* | 0.0000 | 0.0003 | 0.0001 | 0.0001 |
|  |  | *Eubacterium nodatum group* | 0.0001 | 0.0030 | 0.0010 | 0.0024 |
|  |  | *Syntrophococcus* | 0.0004 | 0.0007 | 0.0014 | 0.0061 |
|  | 59 | *Lachnospiraceae NK3A20 group* | 0.0038 | 0.0083 | 0.0136 | 0.0165 |
|  |  | *Dialister* | 0.0004 | 0.0054 | 0.0063 | 0.0040 |
|  |  | *Succinivibrio* | 0.0000 | 0.0001 | 0.0985 | 0.0086 |
|  |  | *Rikenellaceae RC9 gut group* | 0.0435 | 0.0095 | 0.0019 | 0.0086 |
|  |  | *Eubacterium coprostanoligenes group* | 0.0013 | 0.0093 | 0.0025 | 0.0068 |
|  |  | *uncultured Ruminococcaceae* | 0.1297 | 0.0001 | 0.0001 | 0.0001 |
|  |  | *Bacteroidales BS11 gut group* | 0.0160 | 0.0001 | 0.0013 | 0.0002 |
|  |  | *Ruminococcus gauvreauii group* | 0.0032 | 0.0003 | 0.0010 | 0.0012 |
|  |  | *uncultured Veillonellaceae* | 0.0001 | 0.0020 | 0.0004 | 0.0006 |
|  |  | *Syntrophococcus* | 0.0000 | 0.0002 | 0.0006 | 0.0005 |
|  |  | *Lachnospira* | 0.0000 | 0.0026 | 0.0001 | 0.0001 |
|  | 135 | *Prevotella 7* | 0.0072 | 0.0858 | 0.0556 | 0.0326 |
|  |  | *Succiniclasticum* | 0.0026 | 0.0637 | 0.0212 | 0.0414 |
|  |  | *Lachnospiraceae NK3A20 group* | 0.0003 | 0.0055 | 0.0016 | 0.0010 |
|  |  | *Oribacterium* | 0.0007 | 0.0016 | 0.0075 | 0.0274 |
|  |  | *Lachnospiraceae NK4A136 group* | 0.0003 | 0.0143 | 0.0077 | 0.0075 |
|  |  | *Gardnerella* | 0.0021 | 0.0017 | 0.0010 | 0.0006 |
|  |  | *Roseburia* | 0.0001 | 0.0067 | 0.0046 | 0.0079 |
|  |  | *Ruminococcus 2* | 0.0047 | 0.0007 | 0.0012 | 0.0000 |
|  |  | *uncultured Ruminococcaceae* | 0.0513 | 0.0008 | 0.0003 | 0.0008 |
|  |  | *Coprococcus 1* | 0.0003 | 0.0065 | 0.0012 | 0.0017 |
|  |  | *Treponema 2* | 0.0958 | 0.0061 | 0.0003 | 0.0127 |
|  |  | *Bacteroidales S24-7 group* | 0.0184 | 0.0035 | 0.0039 | 0.0002 |
|  |  | *uncultured Lachnospiraceae* | 0.0003 | 0.0037 | 0.0062 | 0.0115 |
|  |  | *Christensenellaceae R-7 group* | 0.0041 | 0.0010 | 0.0016 | 0.0001 |
|  |  | *Bacteroidales UCG-001* | 0.0003 | 0.0048 | 0.0020 | 0.0017 |
|  |  | *Ruminococcaceae UCG-002* | 0.0001 | 0.0057 | 0.0002 | 0.0066 |
|  |  | *Ruminococcus 1* | 0.0005 | 0.0041 | 0.0031 | 0.0011 |
|  |  | *Syntrophococcus* | 0.0003 | 0.0005 | 0.0052 | 0.0007 |
|  |  | *Mobilitalea* | 0.0000 | 0.0016 | 0.0009 | 0.0004 |
|  |  | *Lachnospira* | 0.0000 | 0.0001 | 0.0003 | 0.0004 |
|  | 223 | *Treponema 2* | 0.0001 | 0.0000 | 0.0000 | 0.0000 |
|  |  | *Veillonellaceae ambiguous taxa* | 0.0001 | 0.0056 | 0.0028 | 0.0040 |
|  |  | *uncultured Lachnospiraceae* | 0.0034 | 0.0019 | 0.0018 | 0.0014 |
|  |  | *Megasphaera* | 0.0000 | 0.0001 | 0.0019 | 0.0001 |
|  |  | *Ruminococcaceae UCG-002* | 0.0016 | 0.0007 | 0.0002 | 0.0005 |
|  |  | *Lachnospira* | 0.0007 | 0.0009 | 0.0010 | 0.0018 |
|  |  |  |  |  |  |  |
| LH | 31 | *Succinivibrionaceae UCG-001* | 0.3784 | 0.0130 | 0.1775 | 0.0012 |
|  |  | *Lachnospiraceae NK3A20 group* | 0.0019 | 0.0062 | 0.0202 | 0.0058 |
|  |  | *Erysipelotrichaceae UCG-002* | 0.0333 | 0.0014 | 0.0005 | 0.0000 |
|  |  | *Lachnospiraceae NK4A136 group* | 0.0042 | 0.0154 | 0.0141 | 0.0254 |
|  |  | *Lactobacillus* | 0.0001 | 0.0006 | 0.0011 | 0.0011 |
|  |  | *Coprococcus 1* | 0.0013 | 0.0065 | 0.0029 | 0.0092 |
|  |  | *Ruminococcus gauvreauii group* | 0.0009 | 0.0043 | 0.0037 | 0.0116 |
|  |  | *Eubacterium hallii group* | 0.0017 | 0.0060 | 0.0027 | 0.0078 |
|  |  | *Coriobacteriaceae UCG-003* | 0.0003 | 0.0009 | 0.0027 | 0.0066 |
|  |  | *uncultured Lachnospiraceae* | 0.0001 | 0.0027 | 0.0007 | 0.0040 |
|  |  | *Lachnoclostridium 1* | 0.0084 | 0.0038 | 0.0012 | 0.0001 |
|  |  | *Eubacterium nodatum group* | 0.0002 | 0.0019 | 0.0006 | 0.0036 |
|  |  | *uncultured Veillonellaceae* | 0.0015 | 0.0012 | 0.0011 | 0.0001 |
|  |  | *Mobilitalea* | 0.0005 | 0.0011 | 0.0018 | 0.0016 |
|  | 89 | *Eubacterium coprostanoligenes group* | 0.0044 | 0.0045 | 0.0100 | 0.0185 |
|  |  | *Prevotellaceae UCG-001* | 0.0033 | 0.0105 | 0.0193 | 0.0068 |
|  |  | *Coriobacteriaceae UCG-003* | 0.0002 | 0.0003 | 0.0026 | 0.0029 |
|  |  | *Bacteroidales UCG-001* | 0.0001 | 0.0002 | 0.0012 | 0.0005 |
|  |  | *Ruminococcaceae NK4A214 group* | 0.0005 | 0.0006 | 0.0286 | 0.0344 |
|  |  | *uncultured Veillonellaceae* | 0.0001 | 0.0001 | 0.0004 | 0.0006 |
|  | 483 | *Prevotella 7* | 0.0181 | 0.5360 | 0.0396 | 0.5233 |
|  |  | *S24-7 ambiguous taxa* | 0.0010 | 0.0305 | 0.0026 | 0.0764 |
|  |  | *Lachnospiraceae NK3A20 group* | 0.4838 | 0.0115 | 0.0184 | 0.0486 |
|  |  | *Oribacterium* | 0.0000 | 0.0023 | 0.0006 | 0.0368 |
|  |  | *Eubacterium coprostanoligenes group* | 0.0022 | 0.0090 | 0.0109 | 0.0044 |
|  |  | *Lachnospiraceae NK4A136 group* | 0.0033 | 0.0098 | 0.0516 | 0.0072 |
|  |  | *Lactobacillus* | 0.0002 | 0.0011 | 0.0052 | 0.0010 |
|  |  | *Coprococcus 1* | 0.0012 | 0.0082 | 0.0131 | 0.0048 |
|  |  | *Sphaerochaeta* | 0.0084 | 0.0015 | 0.0039 | 0.0038 |
|  |  | *Eubacterium hallii group* | 0.0005 | 0.0034 | 0.0201 | 0.0033 |
|  |  | *Eubacterium nodatum group* | 0.0007 | 0.0013 | 0.0018 | 0.0044 |
|  |  | *Syntrophococcus* | 0.0001 | 0.0005 | 0.0015 | 0.0024 |
|  |  | *Mobilitalea* | 0.0005 | 0.0017 | 0.0114 | 0.0014 |
|  | 463 | *Oribacterium* | 0.0037 | 0.0377 | 0.0120 | 0.0161 |
|  |  | *Lactobacillus* | 0.0006 | 0.0008 | 0.0010 | 0.0014 |
|  |  | *Gardnerella* | 0.0020 | 0.0070 | 0.0115 | 0.0341 |
|  |  | *Coprococcus 1* | 0.0020 | 0.0120 | 0.0081 | 0.0051 |
|  |  | *Sphaerochaeta* | 0.0000 | 0.0011 | 0.0033 | 0.0087 |
|  |  | *Coriobacteriaceae UCG-003* | 0.0006 | 0.0012 | 0.0021 | 0.0092 |
|  |  | *Ruminococcus 1* | 0.0003 | 0.0014 | 0.0175 | 0.0014 |
|  |  | *Lachnospira* | 0.0006 | 0.0012 | 0.0013 | 0.0014 |
|  |  |  |  |  |  |  |
| HL | 107 | *Prevotella 7* | 0.6603 | 0.6104 | 0.4059 | 0.2888 |
|  |  | *Succiniclasticum* | 0.0237 | 0.0843 | 0.0683 | 0.0506 |
|  |  | *p-2534-18B5 gut group* | 0.0000 | 0.0000 | 0.0001 | 0.0021 |
|  |  | *Rikenellaceae RC9 gut group* | 0.0012 | 0.0022 | 0.0023 | 0.0221 |
|  |  | *Oribacterium* | 0.0102 | 0.0081 | 0.0042 | 0.0034 |
|  |  | *Eubacterium coprostanoligenes group* | 0.0235 | 0.0049 | 0.0064 | 0.0108 |
|  |  | *Gardnerella* | 0.0012 | 0.0087 | 0.0027 | 0.0256 |
|  |  | *Ruminococcus 2* | 0.0000 | 0.0000 | 0.0001 | 0.0008 |
|  |  | *Sphaerochaeta* | 0.0000 | 0.0076 | 0.0007 | 0.0088 |
|  |  | *Prevotellaceae UCG-001* | 0.0002 | 0.0004 | 0.0016 | 0.0018 |
|  |  | *Coriobacteriaceae UCG-003* | 0.0006 | 0.0010 | 0.0058 | 0.0060 |
|  |  | *Veillonellaceae ambiguous taxa* | 0.0233 | 0.0039 | 0.0074 | 0.0016 |
|  |  | *Christensenellaceae R-7 group* | 0.0000 | 0.0000 | 0.0001 | 0.0003 |
|  |  | *Bacteroidales UCG-001* | 0.0000 | 0.0000 | 0.0000 | 0.0026 |
|  |  | *Ruminococcaceae UCG-002* | 0.0001 | 0.0001 | 0.0008 | 0.0014 |
|  |  | *Prevotellaceae UCG-003* | 0.0000 | 0.0000 | 0.0001 | 0.0003 |
|  |  | *Ruminococcus 1* | 0.0002 | 0.0004 | 0.0007 | 0.0019 |
|  | 481 | *Olsenella* | 0.0695 | 0.0289 | 0.0253 | 0.0025 |
|  |  | *Dialister* | 0.0205 | 0.0487 | 0.0436 | 0.0563 |
|  |  | *Gardnerella* | 0.0287 | 0.0211 | 0.0128 | 0.0015 |
|  |  | *Bacteroidales BS11 gut group* | 0.0012 | 0.0001 | 0.0003 | 0.0001 |
|  |  | *Coprococcus 1* | 0.0230 | 0.0030 | 0.0027 | 0.0035 |
|  |  | *Prevotellaceae UCG-001* | 0.0006 | 0.0003 | 0.0002 | 0.0001 |
|  |  | *Eubacterium nodatum group* | 0.0028 | 0.0019 | 0.0018 | 0.0007 |
|  |  | *Lachnospira* | 0.0010 | 0.0001 | 0.0001 | 0.0003 |
|  | 485 | *Eubacterium coprostanoligenes group* | 0.0131 | 0.0126 | 0.0112 | 0.0065 |
|  |  | *Lactobacillus* | 0.0001 | 0.0004 | 0.0138 | 0.0006 |
|  |  | *Gardnerella* | 0.0027 | 0.0124 | 0.0072 | 0.0353 |
|  |  | *Ruminococcaceae UCG-014* | 0.0055 | 0.0063 | 0.0065 | 0.0092 |
|  |  | *uncultured Ruminococcaceae* | 0.0000 | 0.0100 | 0.0001 | 0.0001 |
|  |  | *Sphaerochaeta* | 0.0000 | 0.0076 | 0.0003 | 0.0021 |
|  |  | *Coriobacteriaceae UCG-003* | 0.0004 | 0.0020 | 0.0044 | 0.0070 |
|  |  | *Veillonellaceae ambiguous taxa* | 0.0177 | 0.0050 | 0.0048 | 0.0000 |
|  |  | *Bacteroidales UCG-001* | 0.0001 | 0.0036 | 0.0011 | 0.0004 |
|  |  | *Eubacterium nodatum group* | 0.0010 | 0.0025 | 0.0044 | 0.0029 |
|  |  | *Ruminococcaceae UCG-002* | 0.0000 | 0.0013 | 0.0002 | 0.0001 |
|  |  | *Prevotellaceae UCG-003* | 0.0000 | 0.0004 | 0.0001 | 0.0001 |
|  |  | *Ruminococcus 1* | 0.0014 | 0.0012 | 0.0007 | 0.0005 |
|  |  | *Mobilitalea* | 0.0002 | 0.0015 | 0.0012 | 0.0021 |
|  |  | *Lachnospira* | 0.0003 | 0.0013 | 0.0009 | 0.0010 |
|  | 67 | *Succiniclasticum* | 0.0044 | 0.0007 | 0.0003 | 0.0018 |
|  |  | *Bacteroidales BS11 gut group* | 0.0007 | 0.0001 | 0.0000 | 0.0000 |
|  |  | *Prevotellaceae UCG-001* | 0.0010 | 0.0003 | 0.0004 | 0.0004 |

**Table S4.** Co-variation of microbial profiles and feed efficiency.

| Animal | Microbial profile distance | \|∆FCR\| |
| --- | --- | --- |
| 9 | 0.34^a^ | 1.53^b^ |
| 231 | 0.33 | 1.76 |
| 201 | 0.53 | 0.00 |
| 247 | 0.69 | 13.98 |
| 31 | 0.39 | 18.28 |
| 107 | 0.28 | 2.10 |
| 463 | 0.46 | 1.09 |
| 485 | 0.24 | 1.17 |
| 67 | 0.59 | 1.96 |
| 89 | 0.61 | 1.90 |
| 481 | 0.23 | 11.08 |
| 483 | 0.59 | 0.83 |
| 35 | 0.46 | 15.97 |
| 59 | 0.60 | 2.63 |
| 135 | 0.53 | 11.12 |
| 223 | 0.42 | 1.76 |

^a^ <0.50 indicates similar profiles; ≥0.50 indicates differed profiles.

^b^ <3 indicates minor variation; >10 indicates significant change.

**Table S5.** Correlation between bacteria genus and rumen measurements^a^.

|  | Bacteria genus | Relative abundance | R | *P* |
| --- | --- | --- | --- | --- |
| Acetate | *Ruminococcaceae NK4A214 group* | 0.0020±0.0012 | 0.545 | 0.029 |
|  | *Christensenellaceae R-7 group* | 0.0022±0.0015 | 0.657 | 0.006 |
|  |  |  |  |  |
| Propionate | *Mobilitalea* | 0.0013±0.0002 | 0.551 | 0.027 |
|  | *Eubacterium nodatum group* | 0.0020±0.0003 | 0.585 | 0.017 |
|  | *uncultured Lachnospiraceae* | 0.0027±0.0004 | 0.507 | 0.045 |
|  | *Eubacterium hallii group* | 0.0041±0.0007 | 0.624 | 0.010 |
|  | *Lachnospiraceae NK4A136 group* | 0.0127±0.0021 | 0.527 | 0.036 |
|  |  |  |  |  |
| Butyrate | *Ruminococcaceae NK4A214 group* | 0.0020±0.0012 | 0.634 | 0.008 |
|  |  |  |  |  |
| Isobutyrate | *Ruminococcaceae UCG-002* | 0.0020±0.0005 | 0.672 | 0.004 |
|  | *Sphaerochaeta* | 0.0046±0.0010 | 0.756 | <0.001 |
|  |  |  |  |  |
| Valerate | *Erysipelotrichaceae UCG-002* | 0.0236±0.0098 | 0.597 | 0.015 |
|  | *Coriobacteriaceae UCG-002* | 0.0244±0.0098 | 0.518 | 0.040 |
|  |  |  |  |  |
| Isovalerate | *Ruminococcaceae NK4A214 group* | 0.0020±0.0012 | 0.627 | 0.009 |
|  | *Ruminococcaceae UCG-002* | 0.0020±0.0005 | 0.711 | 0.002 |
|  | *Prevotella 1* | 0.1729±0.0468 | 0.546 | 0.029 |
|  |  |  |  |  |
| Caproate | *Lachnoclostridium 1* | 0.0022±0.0015 | 0.723 | 0.002 |
|  |  |  |  |  |
| NH_3_-N | *Ruminococcaceae NK4A214 group* | 0.0020±0.0012 | 0.791 | <0.001 |
|  | *Christensenellaceae R-7 group* | 0.0022±0.0015 | 0.894 | <0.001 |
|  | *Bacteroidales BS11 gut group* | 0.0062±0.0024 | 0.600 | 0.014 |
|  |  |  |  |  |
| Mean pH | *Prevotellaceae UCG-003* | 0.0018±0.0008 | 0.591 | 0.016 |
|  | *Ruminococcaceae UCG-002* | 0.0020±0.0005 | 0.540 | 0.031 |
|  | *uncultured Lachnospiraceae* | 0.0027±0.0004 | 0.598 | 0.014 |
|  | *Bacteroidales S24-7 group* | 0.0028±0.0009 | 0.520 | 0.039 |
|  | *Treponema 2* | 0.0032±0.0018 | 0.624 | 0.010 |
|  | *Prevotella 1* | 0.1729±0.0468 | 0.545 | 0.029 |

^a^ Only positive correlation between VFA/ammonia was listed to indicate the potential producers.
