# Supplementary material for: Assessment of microbiome changes after rumen transfaunation: implications on improving feed efficiency in beef cattle
Source: Microbiome. 2018 Mar 27;6:62. doi: 10.1186/s40168-018-0447-y (PMC5869788; doi:10.1186/s40168-018-0447-y)
Supplement: Supplementary file 2 — Figure S1. UPGMA clustering of the bacterial profiles by 454 pyrosequencing. Figure S2. UPGMA clustering of the long-term bacterial and archaeal profile changes by PCR-DGGE. Figure S3. UPGMA clustering of the short-term bacterial and archaeal profile changes by PCR-DGGE. Figure S4. The bacterial genera with relative abundance increased after transfaunation. The relative abundance was presented as proportions. The phylotypes were either increased after transfaunation. (ZIP 352 kb) [file 40168_2018_447_MOESM2_ESM.zip › Zhou et al. Microbiome rr fig S4.pdf]

LH *

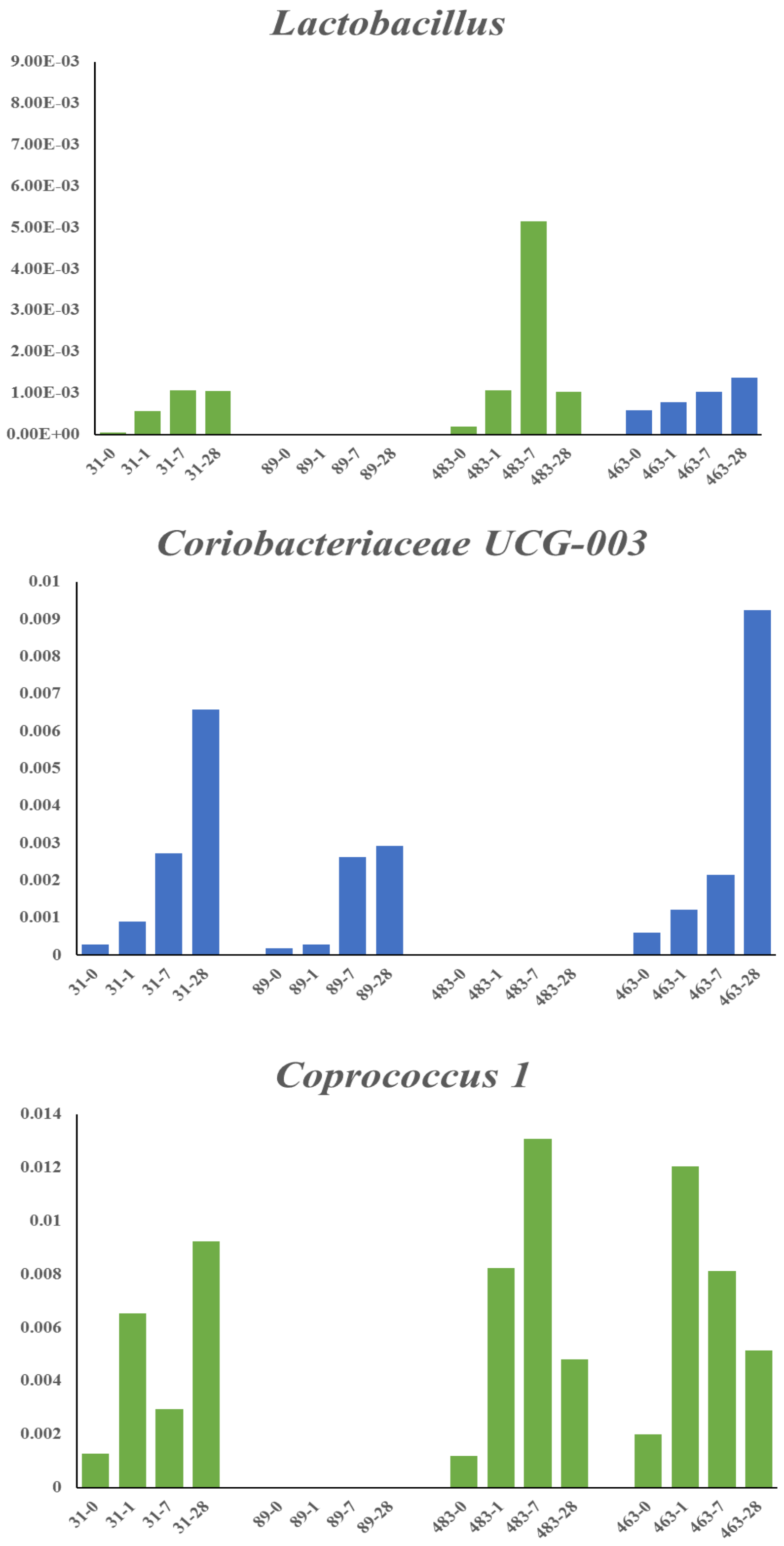

Gradually increased along time

At least 2 fold more abundant after transfaunation

* The data of the animals that doesn’t follow the changing trend was no plotted.

Zhou et al. Figure S4
